# Methyl 3-acet­oxy-3-dehydroxy­ursolate

**DOI:** 10.1107/S1600536809030657

**Published:** 2009-08-08

**Authors:** Nor Hayati Abdullah, Khalijah Awang, Noel F. Thomas, Seik Weng Ng

**Affiliations:** a Medicinal Plant Division, Forest Research Institute Malaysia, 52100 Kepong, Selangor Darul Ehsan, Malaysia; b Department of Chemistry, University of Malaya, 50603 Kuala Lumpur, Malaysia

## Abstract

Four of the five six-membered rings of the title penta­cyclic triterpene, C_33_H_52_O_4_, adopt chair conformations; the fifth, which has a C=C double bond, adopts an approximate envelope conformation.

## Related literature

For the synthesis, see: Ma *et al.* (2005 ▶). The crystal structure of ursolic acid is known from its ethanol solvate; see: Simon *et al.* (1992 ▶). For methyl uroslate-3-bromo­acetate, see: Stout & Stevens (1963 ▶). For methyl ursolate-3-*p*-bromo­benzoate, see: Paton & Paul (1979 ▶).
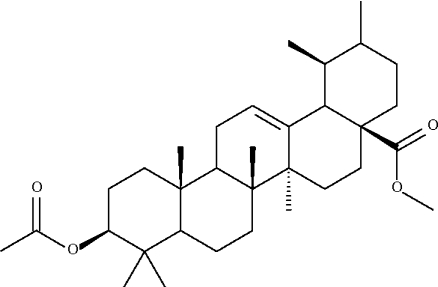


## Experimental

### 

#### Crystal data

C_33_H_52_O_4_
*M* *_r_* = 512.75
Orthorhombic, 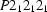

*a* = 6.8245 (1) Å
*b* = 13.4874 (2) Å
*c* = 32.2233 (5) Å
*V* = 2965.99 (8) Å^3^
*Z* = 4
Mo *K*α radiation
μ = 0.07 mm^−1^
*T* = 100 K
0.30 × 0.15 × 0.05 mm

#### Data collection

Bruker SMART APEX diffractometer
Absorption correction: none
28565 measured reflections
3886 independent reflections
3612 reflections with *I* > 2σ(*I*)
*R*_int_ = 0.034

#### Refinement

*R*[*F* ^2^ > 2σ(*F* ^2^)] = 0.036
*wR*(*F* ^2^) = 0.099
*S* = 1.07
3886 reflections
343 parameters
H-atom parameters constrained
Δρ_max_ = 0.35 e Å^−3^
Δρ_min_ = −0.18 e Å^−3^

### 

Data collection: *APEX2* (Bruker, 2008 ▶); cell refinement: *SAINT* (Bruker, 2008 ▶); data reduction: *SAINT*; program(s) used to solve structure: *SHELXS97* (Sheldrick, 2008 ▶); program(s) used to refine structure: *SHELXL97* (Sheldrick, 2008 ▶); molecular graphics: *X-SEED* (Barbour, 2001 ▶); software used to prepare material for publication: *publCIF* (Westrip, 2009 ▶).

## Supplementary Material

Crystal structure: contains datablocks I, global. DOI: 10.1107/S1600536809030657/xu2574sup1.cif

Structure factors: contains datablocks I. DOI: 10.1107/S1600536809030657/xu2574Isup2.hkl

Additional supplementary materials:  crystallographic information; 3D view; checkCIF report

## Figures and Tables

**Fig. 1.**
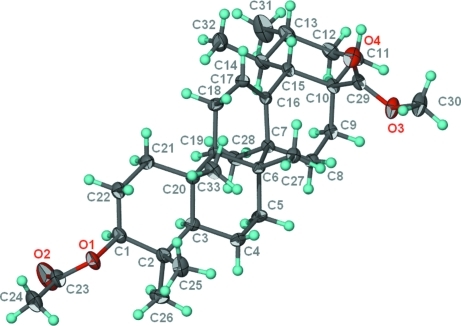
The molecular structure of the methyl ursolate-3-acetate. Displacement ellipsoids are drawn at the 70% probability level, and hydrogen atoms are drawn as spheres of arbitrary radius.

## supplementary crystallographic information

### Experimental

The dried leaves of *Primsatomorismalayana* Ridley (Rubiaceae) (2 kg) were extracted with methanol (10 L). The extract was concentrated and then partitioned with petroleum ether, chloroform and ethyl acetate. The chloroform fraction (35 g) was dissolved in methanol and subjected to column chromatography by using Diaion HP-20 with methanol as the eluent to furnish 200 fractions. After confirming that the fractions contained the same material by TLC analysis, the fractions were pooled into 3 sub-fractions. One sub-fraction was purified by using column chromatography on silica gel (chloroform/methanol10:0 → 9:1) to give ursolic acid (5 g), which was identified acid from its NMR and mass spectra.

The ursolic acid was treated with acetic anhydride and pyridine according to a literature method. The compound was purified by chromatography with a hexane and chloroform system (Ma *et al.*, 2005). Crystals were isolated when the solvent was allowed to evaporate.

### Refinement

The carbon-bound H-atoms were generated geometrically (C—H 0.95–0.99 Å) and were allowed to ride on their parent atoms, with *U*(H) fixed at 1.2–1.5*U*_eq_(C). Friedel pairs were merged.

### Crystal data

**Table:**

C_33_H_52_O_4_	*F*(000) = 1128
*M**_r_* = 512.75	*D*_x_ = 1.148 Mg m^−^^3^
Orthorhombic, *P*2_1_2_1_2_1_	Mo *K*α radiation, λ = 0.71073 Å
Hall symbol: P 2ac 2ab	Cell parameters from 9010 reflections
*a* = 6.8245 (1) Å	θ = 2.4–28.2°
*b* = 13.4874 (2) Å	µ = 0.07 mm^−^^1^
*c* = 32.2233 (5) Å	*T* = 100 K
*V* = 2965.99 (8) Å^3^	Block, colorless
*Z* = 4	0.30 × 0.15 × 0.05 mm

### Data collection

**Table:**

Bruker SMART APEX diffractometer	3612 reflections with *I* > 2σ(*I*)
Radiation source: fine-focus sealed tube	*R*_int_ = 0.034
graphite	θ_max_ = 27.5°, θ_min_ = 1.6°
ω scans	*h* = −8→8
28565 measured reflections	*k* = −17→17
3886 independent reflections	*l* = −41→41

### Refinement

**Table:**

Refinement on *F*^2^	Secondary atom site location: difference Fourier map
Least-squares matrix: full	Hydrogen site location: inferred from neighbouring sites
*R*[*F*^2^ > 2σ(*F*^2^)] = 0.036	H-atom parameters constrained
*wR*(*F*^2^) = 0.099	*w* = 1/[σ^2^(*F*_o_^2^) + (0.0647*P*)^2^ + 0.329*P*] where *P* = (*F*_o_^2^ + 2*F*_c_^2^)/3
*S* = 1.07	(Δ/σ)_max_ = 0.001
3886 reflections	Δρ_max_ = 0.35 e Å^−^^3^
343 parameters	Δρ_min_ = −0.18 e Å^−^^3^
0 restraints	Absolute structure: Friedel pairs were merged.
Primary atom site location: structure-invariant direct methods	

### Fractional atomic coordinates and isotropic or equivalent isotropic displacement parameters (Å^2^)

**Table:**

	*x*	*y*	*z*	*U*_iso_*/*U*_eq_	
O1	0.8683 (2)	0.63811 (9)	0.94880 (4)	0.0230 (3)	
O2	0.5731 (2)	0.69609 (13)	0.93041 (6)	0.0470 (4)	
O3	1.03757 (18)	−0.18360 (9)	0.86981 (4)	0.0233 (3)	
O4	1.20002 (18)	−0.09494 (10)	0.82218 (4)	0.0260 (3)	
C1	0.8406 (3)	0.54575 (12)	0.92596 (5)	0.0186 (3)	
H1	0.7088	0.5471	0.9124	0.022*	
C2	0.8468 (3)	0.45891 (13)	0.95692 (5)	0.0182 (3)	
C3	0.8466 (2)	0.36120 (12)	0.93090 (5)	0.0149 (3)	
H3	0.7162	0.3605	0.9167	0.018*	
C4	0.8493 (3)	0.26628 (12)	0.95708 (5)	0.0180 (3)	
H4A	0.9840	0.2539	0.9672	0.022*	
H4B	0.7628	0.2747	0.9815	0.022*	
C5	0.7799 (3)	0.17749 (12)	0.93146 (5)	0.0170 (3)	
H5A	0.6399	0.1869	0.9245	0.020*	
H5B	0.7903	0.1169	0.9487	0.020*	
C6	0.8959 (2)	0.16124 (12)	0.89087 (5)	0.0130 (3)	
C7	0.7757 (2)	0.09206 (12)	0.86003 (5)	0.0132 (3)	
C8	0.7354 (2)	−0.00924 (12)	0.88113 (5)	0.0157 (3)	
H8A	0.8521	−0.0281	0.8976	0.019*	
H8B	0.6246	−0.0013	0.9007	0.019*	
C9	0.6875 (2)	−0.09377 (12)	0.85118 (5)	0.0161 (3)	
H9A	0.5591	−0.0809	0.8379	0.019*	
H9B	0.6772	−0.1566	0.8669	0.019*	
C10	0.8445 (2)	−0.10472 (12)	0.81746 (5)	0.0153 (3)	
C11	0.8054 (3)	−0.19544 (12)	0.78927 (6)	0.0194 (3)	
H11A	0.9208	−0.2068	0.7713	0.023*	
H11B	0.7873	−0.2550	0.8068	0.023*	
C12	0.6244 (3)	−0.18105 (13)	0.76217 (6)	0.0226 (4)	
H12A	0.5069	−0.1769	0.7801	0.027*	
H12B	0.6088	−0.2393	0.7438	0.027*	
C13	0.6387 (3)	−0.08737 (13)	0.73579 (6)	0.0236 (4)	
H13	0.7532	−0.0950	0.7167	0.028*	
C14	0.6737 (3)	0.00559 (12)	0.76254 (5)	0.0180 (3)	
H14	0.5554	0.0166	0.7803	0.022*	
C15	0.8546 (2)	−0.00871 (12)	0.79156 (5)	0.0148 (3)	
H15	0.9717	−0.0151	0.7731	0.018*	
C16	0.8921 (2)	0.08048 (12)	0.81955 (5)	0.0130 (3)	
C17	1.0181 (2)	0.14961 (12)	0.80750 (5)	0.0158 (3)	
H17	1.0886	0.1375	0.7826	0.019*	
C18	1.0591 (3)	0.24517 (12)	0.82969 (5)	0.0173 (3)	
H18A	1.0380	0.3009	0.8102	0.021*	
H18B	1.1986	0.2462	0.8381	0.021*	
C19	0.9313 (2)	0.26154 (11)	0.86828 (5)	0.0135 (3)	
H19	0.8000	0.2808	0.8571	0.016*	
C20	0.9982 (2)	0.35270 (12)	0.89520 (5)	0.0144 (3)	
C21	0.9818 (3)	0.44612 (12)	0.86787 (5)	0.0189 (3)	
H21A	1.0873	0.4448	0.8468	0.023*	
H21B	0.8549	0.4447	0.8530	0.023*	
C22	0.9963 (3)	0.54291 (12)	0.89246 (5)	0.0206 (4)	
H22A	1.1277	0.5482	0.9053	0.025*	
H22B	0.9788	0.6000	0.8735	0.025*	
C23	0.7261 (3)	0.70689 (15)	0.94762 (6)	0.0280 (4)	
C24	0.7851 (4)	0.79780 (15)	0.97118 (7)	0.0381 (5)	
H24A	0.6719	0.8420	0.9740	0.057*	
H24B	0.8899	0.8322	0.9562	0.057*	
H24C	0.8322	0.7787	0.9988	0.057*	
C25	1.0202 (3)	0.46737 (14)	0.98688 (6)	0.0256 (4)	
H25A	0.9960	0.5213	1.0066	0.038*	
H25B	1.1401	0.4813	0.9712	0.038*	
H25C	1.0357	0.4049	1.0020	0.038*	
C26	0.6580 (3)	0.46347 (15)	0.98272 (6)	0.0260 (4)	
H26A	0.6476	0.5286	0.9960	0.039*	
H26B	0.6611	0.4117	1.0041	0.039*	
H26C	0.5447	0.4530	0.9646	0.039*	
C27	1.0929 (2)	0.11123 (12)	0.90219 (5)	0.0166 (3)	
H27A	1.1746	0.1581	0.9177	0.025*	
H27B	1.1610	0.0914	0.8767	0.025*	
H27C	1.0679	0.0525	0.9193	0.025*	
C28	0.5736 (2)	0.13716 (12)	0.84835 (5)	0.0167 (3)	
H28A	0.4877	0.0848	0.8377	0.025*	
H28B	0.5916	0.1879	0.8269	0.025*	
H28C	0.5142	0.1672	0.8730	0.025*	
C29	1.0468 (3)	−0.12439 (12)	0.83603 (5)	0.0173 (3)	
C30	1.2249 (3)	−0.20894 (15)	0.88788 (7)	0.0288 (4)	
H30A	1.2043	−0.2512	0.9122	0.043*	
H30B	1.2930	−0.1482	0.8963	0.043*	
H30C	1.3043	−0.2445	0.8674	0.043*	
C31	0.4540 (4)	−0.07768 (16)	0.70914 (8)	0.0413 (6)	
H31A	0.4315	−0.1398	0.6941	0.062*	
H31B	0.4711	−0.0235	0.6892	0.062*	
H31C	0.3412	−0.0636	0.7270	0.062*	
C32	0.7024 (3)	0.09685 (14)	0.73502 (5)	0.0263 (4)	
H32A	0.5835	0.1083	0.7187	0.039*	
H32B	0.8135	0.0858	0.7163	0.039*	
H32C	0.7285	0.1549	0.7525	0.039*	
C33	1.2140 (3)	0.34623 (13)	0.91040 (6)	0.0202 (4)	
H33A	1.2638	0.4131	0.9158	0.030*	
H33B	1.2945	0.3145	0.8890	0.030*	
H33C	1.2198	0.3070	0.9360	0.030*	

### Atomic displacement parameters (Å^2^)

**Table:**

	*U*^11^	*U*^22^	*U*^33^	*U*^12^	*U*^13^	*U*^23^
O1	0.0271 (7)	0.0157 (6)	0.0262 (6)	0.0066 (5)	−0.0052 (5)	−0.0058 (5)
O2	0.0382 (9)	0.0427 (9)	0.0601 (10)	0.0214 (8)	−0.0182 (8)	−0.0115 (8)
O3	0.0138 (6)	0.0226 (6)	0.0335 (7)	−0.0004 (5)	−0.0040 (5)	0.0111 (5)
O4	0.0120 (6)	0.0300 (7)	0.0360 (7)	0.0001 (5)	0.0007 (5)	0.0086 (6)
C1	0.0201 (8)	0.0162 (8)	0.0194 (8)	0.0032 (7)	−0.0043 (7)	−0.0043 (6)
C2	0.0202 (8)	0.0180 (8)	0.0164 (7)	0.0022 (7)	−0.0036 (6)	−0.0038 (6)
C3	0.0133 (7)	0.0177 (8)	0.0137 (7)	−0.0002 (6)	−0.0020 (6)	−0.0023 (6)
C4	0.0213 (8)	0.0201 (8)	0.0127 (7)	0.0002 (7)	−0.0005 (6)	0.0000 (6)
C5	0.0181 (8)	0.0191 (8)	0.0139 (7)	−0.0029 (7)	0.0037 (6)	0.0006 (6)
C6	0.0113 (7)	0.0148 (7)	0.0130 (7)	−0.0007 (6)	0.0005 (6)	0.0012 (6)
C7	0.0107 (7)	0.0136 (7)	0.0154 (7)	−0.0012 (6)	0.0003 (6)	−0.0008 (6)
C8	0.0142 (8)	0.0156 (7)	0.0173 (7)	−0.0021 (6)	0.0028 (6)	0.0011 (6)
C9	0.0118 (7)	0.0138 (7)	0.0226 (8)	−0.0017 (6)	0.0005 (6)	0.0011 (6)
C10	0.0112 (7)	0.0135 (7)	0.0212 (8)	0.0001 (6)	0.0002 (6)	−0.0008 (6)
C11	0.0173 (8)	0.0151 (8)	0.0258 (8)	0.0002 (7)	0.0015 (7)	−0.0032 (7)
C12	0.0201 (8)	0.0196 (8)	0.0280 (9)	−0.0004 (7)	−0.0019 (7)	−0.0087 (7)
C13	0.0247 (9)	0.0220 (8)	0.0241 (8)	0.0043 (8)	−0.0052 (7)	−0.0084 (7)
C14	0.0171 (8)	0.0189 (8)	0.0180 (8)	0.0051 (7)	−0.0038 (7)	−0.0051 (6)
C15	0.0124 (7)	0.0141 (7)	0.0179 (7)	0.0018 (6)	−0.0006 (6)	−0.0011 (6)
C16	0.0115 (7)	0.0145 (7)	0.0131 (7)	0.0027 (6)	−0.0016 (6)	−0.0003 (6)
C17	0.0162 (8)	0.0173 (8)	0.0139 (7)	0.0011 (7)	0.0016 (6)	−0.0008 (6)
C18	0.0190 (8)	0.0166 (7)	0.0163 (7)	−0.0049 (7)	0.0051 (7)	−0.0004 (6)
C19	0.0127 (7)	0.0143 (7)	0.0136 (7)	−0.0012 (6)	0.0006 (6)	0.0000 (6)
C20	0.0138 (7)	0.0152 (7)	0.0140 (7)	−0.0011 (6)	−0.0009 (6)	−0.0010 (6)
C21	0.0239 (8)	0.0152 (7)	0.0176 (8)	−0.0023 (7)	0.0016 (7)	−0.0008 (6)
C22	0.0257 (9)	0.0139 (7)	0.0223 (8)	−0.0009 (7)	0.0003 (7)	−0.0007 (6)
C23	0.0335 (11)	0.0246 (9)	0.0259 (9)	0.0117 (9)	0.0013 (8)	0.0008 (7)
C24	0.0532 (14)	0.0212 (9)	0.0398 (11)	0.0116 (10)	0.0032 (11)	−0.0044 (9)
C25	0.0317 (10)	0.0211 (9)	0.0241 (9)	0.0026 (8)	−0.0143 (8)	−0.0051 (7)
C26	0.0303 (10)	0.0269 (10)	0.0208 (8)	0.0014 (8)	0.0049 (7)	−0.0077 (7)
C27	0.0135 (7)	0.0177 (8)	0.0185 (7)	0.0003 (6)	−0.0024 (6)	0.0012 (6)
C28	0.0109 (7)	0.0184 (8)	0.0209 (8)	0.0008 (6)	−0.0002 (6)	−0.0031 (6)
C29	0.0141 (8)	0.0137 (7)	0.0241 (8)	0.0007 (6)	−0.0006 (6)	−0.0005 (6)
C30	0.0181 (8)	0.0290 (9)	0.0395 (10)	0.0015 (8)	−0.0091 (8)	0.0122 (8)
C31	0.0456 (13)	0.0302 (10)	0.0481 (13)	0.0059 (10)	−0.0271 (11)	−0.0149 (10)
C32	0.0353 (10)	0.0229 (9)	0.0206 (8)	0.0057 (8)	−0.0093 (8)	−0.0014 (7)
C33	0.0138 (8)	0.0193 (8)	0.0276 (8)	−0.0004 (7)	−0.0022 (7)	−0.0045 (7)

### Geometric parameters (Å, °)

**Table:**

O1—C23	1.343 (2)	C14—H14	1.0000
O1—C1	1.459 (2)	C15—C16	1.525 (2)
O2—C23	1.191 (3)	C15—H15	1.0000
O3—C29	1.351 (2)	C16—C17	1.326 (2)
O3—C30	1.446 (2)	C17—C18	1.500 (2)
O4—C29	1.205 (2)	C17—H17	0.9500
C1—C22	1.515 (2)	C18—C19	1.535 (2)
C1—C2	1.539 (2)	C18—H18A	0.9900
C1—H1	1.0000	C18—H18B	0.9900
C2—C25	1.532 (2)	C19—C20	1.572 (2)
C2—C26	1.535 (3)	C19—H19	1.0000
C2—C3	1.562 (2)	C20—C21	1.541 (2)
C3—C4	1.533 (2)	C20—C33	1.554 (2)
C3—C20	1.552 (2)	C21—C22	1.530 (2)
C3—H3	1.0000	C21—H21A	0.9900
C4—C5	1.530 (2)	C21—H21B	0.9900
C4—H4A	0.9900	C22—H22A	0.9900
C4—H4B	0.9900	C22—H22B	0.9900
C5—C6	1.544 (2)	C23—C24	1.497 (3)
C5—H5A	0.9900	C24—H24A	0.9800
C5—H5B	0.9900	C24—H24B	0.9800
C6—C27	1.548 (2)	C24—H24C	0.9800
C6—C19	1.555 (2)	C25—H25A	0.9800
C6—C7	1.591 (2)	C25—H25B	0.9800
C7—C16	1.535 (2)	C25—H25C	0.9800
C7—C8	1.551 (2)	C26—H26A	0.9800
C7—C28	1.554 (2)	C26—H26B	0.9800
C8—C9	1.529 (2)	C26—H26C	0.9800
C8—H8A	0.9900	C27—H27A	0.9800
C8—H8B	0.9900	C27—H27B	0.9800
C9—C10	1.533 (2)	C27—H27C	0.9800
C9—H9A	0.9900	C28—H28A	0.9800
C9—H9B	0.9900	C28—H28B	0.9800
C10—C29	1.528 (2)	C28—H28C	0.9800
C10—C15	1.542 (2)	C30—H30A	0.9800
C10—C11	1.547 (2)	C30—H30B	0.9800
C11—C12	1.525 (2)	C30—H30C	0.9800
C11—H11A	0.9900	C31—H31A	0.9800
C11—H11B	0.9900	C31—H31B	0.9800
C12—C13	1.526 (3)	C31—H31C	0.9800
C12—H12A	0.9900	C32—H32A	0.9800
C12—H12B	0.9900	C32—H32B	0.9800
C13—C31	1.530 (3)	C32—H32C	0.9800
C13—C14	1.540 (2)	C33—H33A	0.9800
C13—H13	1.0000	C33—H33B	0.9800
C14—C32	1.530 (2)	C33—H33C	0.9800
C14—C15	1.561 (2)		
			
C23—O1—C1	118.81 (15)	C15—C16—C7	119.72 (14)
C29—O3—C30	115.02 (14)	C16—C17—C18	125.83 (15)
O1—C1—C22	106.87 (14)	C16—C17—H17	117.1
O1—C1—C2	108.62 (13)	C18—C17—H17	117.1
C22—C1—C2	115.04 (14)	C17—C18—C19	113.80 (13)
O1—C1—H1	108.7	C17—C18—H18A	108.8
C22—C1—H1	108.7	C19—C18—H18A	108.8
C2—C1—H1	108.7	C17—C18—H18B	108.8
C25—C2—C26	107.72 (14)	C19—C18—H18B	108.8
C25—C2—C1	111.91 (15)	H18A—C18—H18B	107.7
C26—C2—C1	107.31 (15)	C18—C19—C6	110.02 (12)
C25—C2—C3	113.71 (14)	C18—C19—C20	113.22 (13)
C26—C2—C3	108.88 (14)	C6—C19—C20	117.85 (12)
C1—C2—C3	107.11 (12)	C18—C19—H19	104.8
C4—C3—C20	109.75 (13)	C6—C19—H19	104.8
C4—C3—C2	114.15 (12)	C20—C19—H19	104.8
C20—C3—C2	117.36 (14)	C21—C20—C3	108.33 (13)
C4—C3—H3	104.7	C21—C20—C33	107.15 (14)
C20—C3—H3	104.7	C3—C20—C33	113.73 (13)
C2—C3—H3	104.7	C21—C20—C19	107.63 (12)
C5—C4—C3	110.66 (13)	C3—C20—C19	105.84 (13)
C5—C4—H4A	109.5	C33—C20—C19	113.90 (13)
C3—C4—H4A	109.5	C22—C21—C20	113.38 (13)
C5—C4—H4B	109.5	C22—C21—H21A	108.9
C3—C4—H4B	109.5	C20—C21—H21A	108.9
H4A—C4—H4B	108.1	C22—C21—H21B	108.9
C4—C5—C6	114.17 (14)	C20—C21—H21B	108.9
C4—C5—H5A	108.7	H21A—C21—H21B	107.7
C6—C5—H5A	108.7	C1—C22—C21	110.20 (14)
C4—C5—H5B	108.7	C1—C22—H22A	109.6
C6—C5—H5B	108.7	C21—C22—H22A	109.6
H5A—C5—H5B	107.6	C1—C22—H22B	109.6
C5—C6—C27	107.91 (12)	C21—C22—H22B	109.6
C5—C6—C19	110.64 (12)	H22A—C22—H22B	108.1
C27—C6—C19	110.76 (13)	O2—C23—O1	124.20 (19)
C5—C6—C7	110.36 (12)	O2—C23—C24	124.88 (19)
C27—C6—C7	109.84 (12)	O1—C23—C24	110.92 (18)
C19—C6—C7	107.34 (12)	C23—C24—H24A	109.5
C16—C7—C8	112.01 (13)	C23—C24—H24B	109.5
C16—C7—C28	107.05 (12)	H24A—C24—H24B	109.5
C8—C7—C28	107.09 (13)	C23—C24—H24C	109.5
C16—C7—C6	108.88 (12)	H24A—C24—H24C	109.5
C8—C7—C6	109.53 (12)	H24B—C24—H24C	109.5
C28—C7—C6	112.29 (12)	C2—C25—H25A	109.5
C9—C8—C7	114.71 (13)	C2—C25—H25B	109.5
C9—C8—H8A	108.6	H25A—C25—H25B	109.5
C7—C8—H8A	108.6	C2—C25—H25C	109.5
C9—C8—H8B	108.6	H25A—C25—H25C	109.5
C7—C8—H8B	108.6	H25B—C25—H25C	109.5
H8A—C8—H8B	107.6	C2—C26—H26A	109.5
C8—C9—C10	111.69 (13)	C2—C26—H26B	109.5
C8—C9—H9A	109.3	H26A—C26—H26B	109.5
C10—C9—H9A	109.3	C2—C26—H26C	109.5
C8—C9—H9B	109.3	H26A—C26—H26C	109.5
C10—C9—H9B	109.3	H26B—C26—H26C	109.5
H9A—C9—H9B	107.9	C6—C27—H27A	109.5
C29—C10—C9	111.76 (13)	C6—C27—H27B	109.5
C29—C10—C15	108.50 (13)	H27A—C27—H27B	109.5
C9—C10—C15	109.50 (13)	C6—C27—H27C	109.5
C29—C10—C11	104.39 (13)	H27A—C27—H27C	109.5
C9—C10—C11	111.83 (13)	H27B—C27—H27C	109.5
C15—C10—C11	110.73 (13)	C7—C28—H28A	109.5
C12—C11—C10	112.04 (14)	C7—C28—H28B	109.5
C12—C11—H11A	109.2	H28A—C28—H28B	109.5
C10—C11—H11A	109.2	C7—C28—H28C	109.5
C12—C11—H11B	109.2	H28A—C28—H28C	109.5
C10—C11—H11B	109.2	H28B—C28—H28C	109.5
H11A—C11—H11B	107.9	O4—C29—O3	122.26 (16)
C11—C12—C13	111.89 (15)	O4—C29—C10	125.60 (15)
C11—C12—H12A	109.2	O3—C29—C10	112.09 (14)
C13—C12—H12A	109.2	O3—C30—H30A	109.5
C11—C12—H12B	109.2	O3—C30—H30B	109.5
C13—C12—H12B	109.2	H30A—C30—H30B	109.5
H12A—C12—H12B	107.9	O3—C30—H30C	109.5
C12—C13—C31	109.33 (17)	H30A—C30—H30C	109.5
C12—C13—C14	111.85 (14)	H30B—C30—H30C	109.5
C31—C13—C14	111.87 (15)	C13—C31—H31A	109.5
C12—C13—H13	107.9	C13—C31—H31B	109.5
C31—C13—H13	107.9	H31A—C31—H31B	109.5
C14—C13—H13	107.9	C13—C31—H31C	109.5
C32—C14—C13	110.53 (14)	H31A—C31—H31C	109.5
C32—C14—C15	110.22 (14)	H31B—C31—H31C	109.5
C13—C14—C15	110.96 (13)	C14—C32—H32A	109.5
C32—C14—H14	108.4	C14—C32—H32B	109.5
C13—C14—H14	108.3	H32A—C32—H32B	109.5
C15—C14—H14	108.3	C14—C32—H32C	109.5
C16—C15—C10	110.48 (12)	H32A—C32—H32C	109.5
C16—C15—C14	112.93 (13)	H32B—C32—H32C	109.5
C10—C15—C14	113.12 (13)	C20—C33—H33A	109.5
C16—C15—H15	106.6	C20—C33—H33B	109.5
C10—C15—H15	106.6	H33A—C33—H33B	109.5
C14—C15—H15	106.6	C20—C33—H33C	109.5
C17—C16—C15	119.36 (14)	H33A—C33—H33C	109.5
C17—C16—C7	120.84 (14)	H33B—C33—H33C	109.5
			
C23—O1—C1—C22	115.74 (17)	C13—C14—C15—C16	−178.48 (14)
C23—O1—C1—C2	−119.58 (17)	C32—C14—C15—C10	−174.81 (13)
O1—C1—C2—C25	−46.91 (19)	C13—C14—C15—C10	−52.05 (18)
C22—C1—C2—C25	72.79 (18)	C10—C15—C16—C17	137.94 (15)
O1—C1—C2—C26	71.06 (17)	C14—C15—C16—C17	−94.23 (18)
C22—C1—C2—C26	−169.24 (14)	C10—C15—C16—C7	−45.42 (19)
O1—C1—C2—C3	−172.16 (14)	C14—C15—C16—C7	82.41 (17)
C22—C1—C2—C3	−52.46 (19)	C8—C7—C16—C17	−147.40 (15)
C25—C2—C3—C4	56.7 (2)	C28—C7—C16—C17	95.51 (18)
C26—C2—C3—C4	−63.39 (18)	C6—C7—C16—C17	−26.1 (2)
C1—C2—C3—C4	−179.13 (15)	C8—C7—C16—C15	36.01 (19)
C25—C2—C3—C20	−73.76 (18)	C28—C7—C16—C15	−81.08 (16)
C26—C2—C3—C20	166.14 (14)	C6—C7—C16—C15	157.30 (13)
C1—C2—C3—C20	50.40 (18)	C15—C16—C17—C18	174.87 (15)
C20—C3—C4—C5	−65.11 (17)	C7—C16—C17—C18	−1.7 (3)
C2—C3—C4—C5	160.77 (15)	C16—C17—C18—C19	−3.2 (2)
C3—C4—C5—C6	55.33 (19)	C17—C18—C19—C6	36.47 (18)
C4—C5—C6—C27	77.97 (17)	C17—C18—C19—C20	170.68 (13)
C4—C5—C6—C19	−43.35 (18)	C5—C6—C19—C18	175.97 (13)
C4—C5—C6—C7	−162.01 (13)	C27—C6—C19—C18	56.35 (16)
C5—C6—C7—C16	178.09 (13)	C7—C6—C19—C18	−63.56 (16)
C27—C6—C7—C16	−63.05 (15)	C5—C6—C19—C20	44.13 (19)
C19—C6—C7—C16	57.44 (15)	C27—C6—C19—C20	−75.49 (17)
C5—C6—C7—C8	−59.12 (16)	C7—C6—C19—C20	164.60 (13)
C27—C6—C7—C8	59.74 (16)	C4—C3—C20—C21	176.26 (13)
C19—C6—C7—C8	−179.77 (12)	C2—C3—C20—C21	−51.26 (18)
C5—C6—C7—C28	59.72 (16)	C4—C3—C20—C33	−64.72 (17)
C27—C6—C7—C28	178.58 (12)	C2—C3—C20—C33	67.75 (18)
C19—C6—C7—C28	−60.93 (15)	C4—C3—C20—C19	61.07 (15)
C16—C7—C8—C9	−38.61 (19)	C2—C3—C20—C19	−166.46 (13)
C28—C7—C8—C9	78.46 (16)	C18—C19—C20—C21	61.10 (17)
C6—C7—C8—C9	−159.53 (13)	C6—C19—C20—C21	−168.51 (13)
C7—C8—C9—C10	53.32 (19)	C18—C19—C20—C3	176.77 (13)
C8—C9—C10—C29	58.72 (17)	C6—C19—C20—C3	−52.84 (17)
C8—C9—C10—C15	−61.54 (17)	C18—C19—C20—C33	−57.54 (18)
C8—C9—C10—C11	175.34 (13)	C6—C19—C20—C33	72.85 (17)
C29—C10—C11—C12	−170.26 (14)	C3—C20—C21—C22	52.64 (18)
C9—C10—C11—C12	68.73 (18)	C33—C20—C21—C22	−70.44 (17)
C15—C10—C11—C12	−53.68 (18)	C19—C20—C21—C22	166.66 (15)
C10—C11—C12—C13	56.19 (19)	O1—C1—C22—C21	178.07 (13)
C11—C12—C13—C31	179.52 (15)	C2—C1—C22—C21	57.41 (19)
C11—C12—C13—C14	−56.0 (2)	C20—C21—C22—C1	−56.75 (19)
C12—C13—C14—C32	175.81 (16)	C1—O1—C23—O2	3.3 (3)
C31—C13—C14—C32	−61.2 (2)	C1—O1—C23—C24	−177.37 (15)
C12—C13—C14—C15	53.2 (2)	C30—O3—C29—O4	−0.1 (3)
C31—C13—C14—C15	176.25 (16)	C30—O3—C29—C10	177.34 (15)
C29—C10—C15—C16	−66.28 (16)	C9—C10—C29—O4	−145.43 (17)
C9—C10—C15—C16	55.95 (17)	C15—C10—C29—O4	−24.6 (2)
C11—C10—C15—C16	179.71 (13)	C11—C10—C29—O4	93.5 (2)
C29—C10—C15—C14	166.00 (13)	C9—C10—C29—O3	37.18 (19)
C9—C10—C15—C14	−71.78 (16)	C15—C10—C29—O3	158.02 (13)
C11—C10—C15—C14	51.99 (17)	C11—C10—C29—O3	−83.87 (16)
C32—C14—C15—C16	58.75 (17)		
